# Supplementary material for: A vertical silicon-graphene-germanium transistor
Source: Nat Commun. 2019 Oct 25;10:4873. doi: 10.1038/s41467-019-12814-1 (PMC6814790; doi:10.1038/s41467-019-12814-1)
Supplement: Supplementary file 1 — Supplementary Information [file 41467_2019_12814_MOESM1_ESM.pdf]

Supplementary Information

A vertical silicon-graphene-germanium transistor

Liu *et al*.

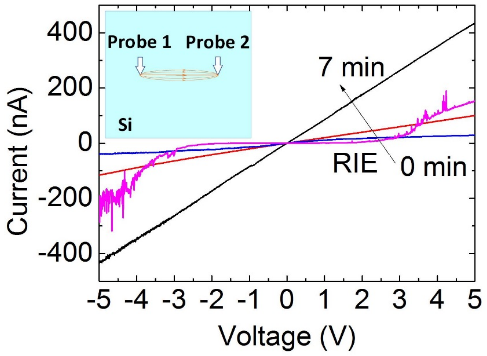

Supplementary Fig. 1 Contact characteristics between the probe and surface of SOI wafers with different RIE etching times from 0 to 7 min. Two probes directly touched the surface of the top Si layer of a SOI wafer. The contact characteristics change from the Schottky type to the Ohmic type with the increasing RIE time. Inset: illustrations of the measurement setup.

In the RIE process of the transistor fabrication, the etching of the Si surface began after the complete etching away of the photoresist that was covering it. Sufficient RIE not only gave a patterned Si but also gave an Ohmic contact between the deposited Au and the top surface of Si. To elucidate this point, the effect of RIE was studied. The same SOI wafers as the Si membrane fabrication were etched by RIE directly with different times of 0, 3, 5 and 7 min. In the measurement, two probes directly touched the etched surface of the top Si layer of the SOI wafers with a distance of ~50 μm between them. As the RIE time increases, the contact between the probe and the surface changes from the Schottky type to the Ohmic type. The sufficient etching damages the Si surface where defect-assisted leakage and high recombination rates lead to the Ohmic contact[1].

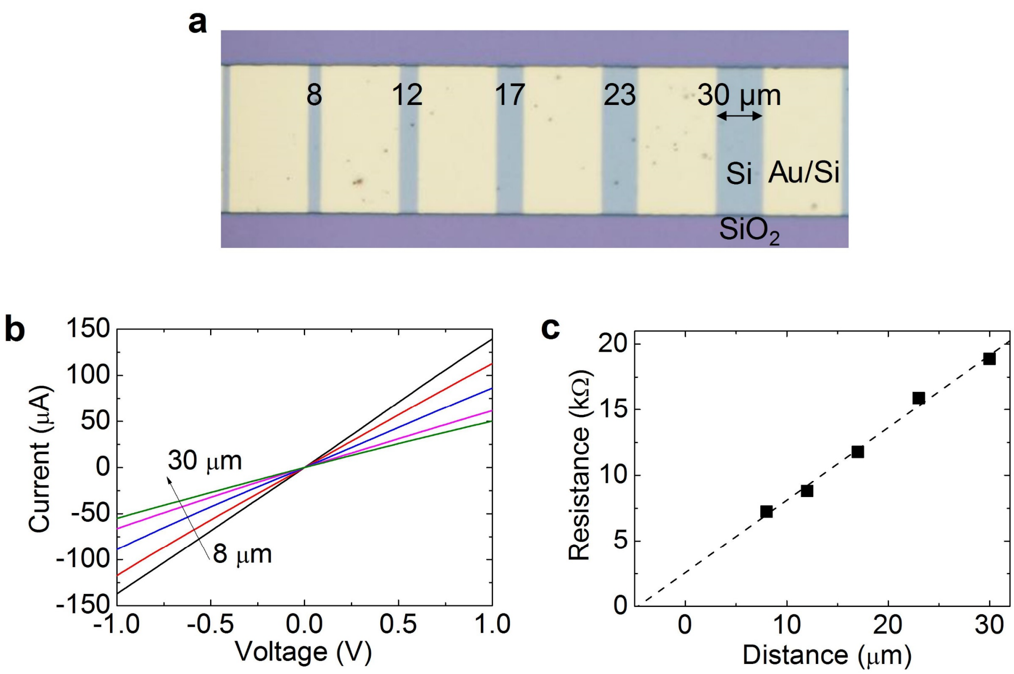

Supplementary Fig. 2 Resistivity measurements of the RIE-etched Si layer on a SOI wafer based on a transfer length method. **a** Optical micrograph of a Si strip with deposited Au electrodes in different distances of 8, 12, 17, 23 and 30 μm. **b** I-V characteristics between adjacent Au electrodes. **c** Relationship of the resistance and distance.

A transfer length method test[1] was carried out to further investigate the Ohmic contact between Au and RIE-etched Si surface. The I-V characteristics between adjacent Au electrodes indicate an Ohmic contact. The resistance and distance have a linear relationship as fitted (dash line in Supplementary Fig. 2c). The slope equals to $\rho/(Wt)$, where ρ is the resistivity of Si, W is the width of Au electrode (93 μm), and t is the thickness of the Si trip (880 nm) (Supplementary Fig. 16). ρ was fitted to be 4.5 Ω cm which is similar to the original SOI wafer (1-6 Ω cm), indicating the RIE processes does not change the doping concentration of Si obviously. The intercept on Y-axis (when distance becomes 0) equals to $2R_c$ where R_c is the contact resistance between Au

and the Si strip (1.3 kΩ). The intercept on X-axis (when resistance becomes 0) equals to $-2L_t$, where L_t is the transfer length. The specific contact resistivity ρ_c was calculated to be $\rho_c = L_t^2 \cdot (\rho/t) = 2.72\times10^{-3}$ Ω cm^2. With the area of the Si membrane ($S = 6.76\times10^{-6}$ cm^2), the contact resistance between Au and the Si membrane in the transistor was estimated to be $\rho_c/S = 402$ Ω, which is consistent with the total series resistance (426 Ω) of the Si-Gr emitter at −5 V (Fig. 2a).

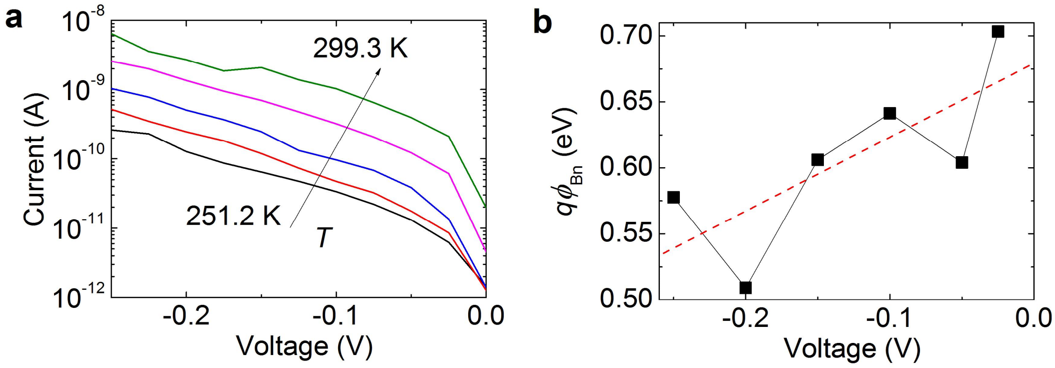

Supplementary Fig. 3 Temperature-dependent I-V characteristics of the Si-Gr junction. **a** The temperature dependence of the current indicates a Schottky behavior. The temperatures are 251.2, 261.7, 273.2, 285.6 and 299.3 K. **b** Schottky barrier height estimation at V=0 by an extrapolation method.

To elucidate the nature of the Si-Gr Schottky junction, temperature-dependent characteristics were measured and the on-current shows an obvious temperature dependence (Supplementary Fig. 3a), which is the feature of the thermionic emission of a Schottky junction. In contrast, the current of a tunnel junction is temperature independent[2]. To fit the Schottky barrier height, firstly, the currents at a small forward bias of 0.1 V (–0.1 V at n-Si side) were considered to avoid the effect of series resistance. The following model was used to fit the relationship between current and temperature: $\ln(I/T^2)=C-q(\phi_{Bn}-V/\eta)/k\cdot(1/T)$, where I is current, T is temperature, C is a constant, q is elementary charge, $q\phi_{Bn}$ is Schottky barrier height, V is voltage, η is the ideality factor of 1.85 (Supplementary Fig. 15), and k is the Boltzmann constant[2]. $q\phi_{Bn}$ was fitted to be ~0.64 eV at room temperature (Fig. 2b). As the temperature decreases, the thermal emission becomes weaker and the tunnel current starts to emerge which shows a weak temperature dependence (Fig. 2b).

Because of the image-force lowering and recombination at the interface, the equivalent potential barrier height is voltage dependent[2]. Using an extrapolation method, the barriers of the Si-Gr junction at V=0 are determined as 0.68 eV (Supplementary Fig. 3b).

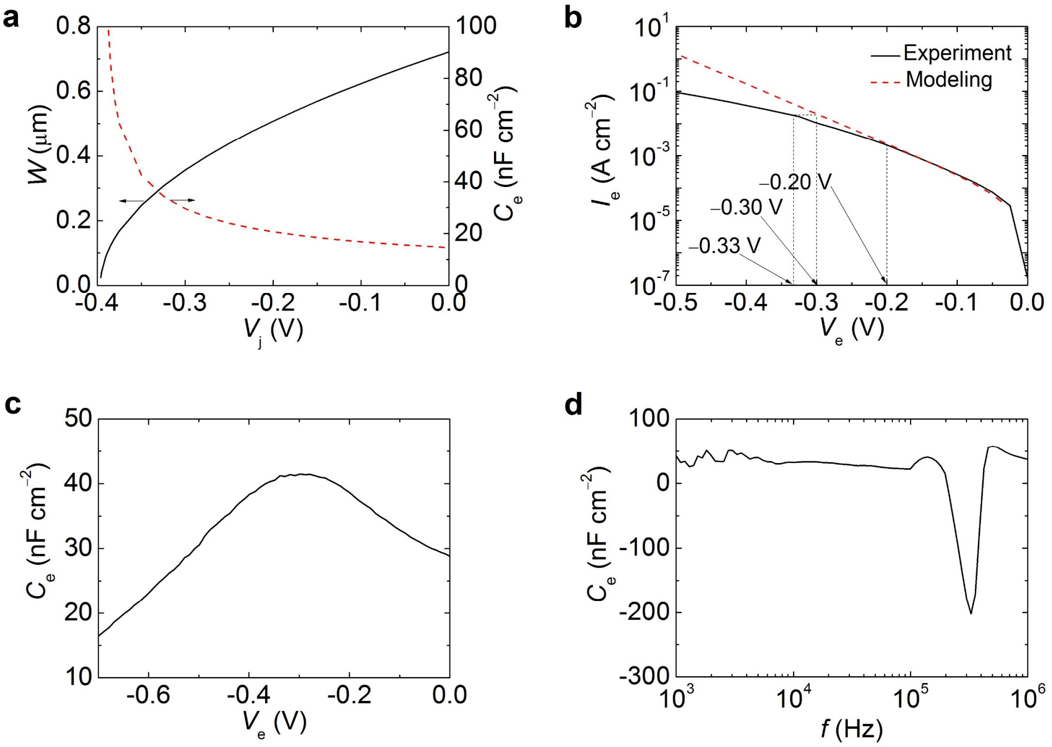

Supplementary Fig. 4 Capacitance of the Si-Gr Schottky emitter. **a** The width of the depletion region of Si (W) and the emitter capacitance (C_e) calculated from the plate-capacitor model, with an applied voltage V_j on the space charge region. **b** The I-V characteristics of the emitter (experiment) was fitted by a Schottky junction model without series resistance (modeling), to determine the voltage on the emitter (0.33 V) when peak capacitance is achieved. **c** The C-V characteristic of the Si-Gr emitter obtained from experiments at a frequency of 100 kHz. **d** The C-f characteristic of the Si-Gr emitter at a forward bias of –0.30 V.

The capacitance of the Gr-Si junction was analyzed by considering the fact that the space charge region tends to vanish as the applied voltage increases. When the applied voltage on the space charge region V_j is small, the capacitance can be calculated from the plate-capacitor model[2] as $C_e=\varepsilon\varepsilon_0/W$, $W=(2\varepsilon\varepsilon_0/qN_d\cdot(\psi_{bi}-V_j-V_t))^{0.5}$, $\psi_{bi}=\phi_{Bn}-V_n$, $V_n=V_t\cdot\ln(N_c/N_d)$, where ε is the relative dielectric constant, ε_0 is the permittivity in

vacuum, W is the width of the depletion region for Si, q is elementary charge, N_d is the doping concentration of Si of about 1×10^{15} cm^{-3}, ψ_{bi} is the potential barrier height at the Si side when no bias is applied, V_t is the thermal voltage, $q\phi_{Bn}$ is the Schottky barrier height of ~0.68 eV, V_n is the Fermi potential of the Si, and N_c is the effective density of states in conduction band of Si. ψ_{bi} is ~0.42 V as calculated. The width of the depletion region (W) is ~722 nm, and the emitter junction capacitance is ~15 nF cm^{-2} at V_j =0 (Supplementary Fig. 4a).

However, the above model encounters a difficulty when W approaches 0, resulting an infinite capacitance and thus infinite free-carrier charges. This violates the fact that the space charge region vanishes and thus the charges stored inside also vanish[3-7]. In fact, as experiments, simulations and modelings show, instead of increasing rapidly towards infinite, the capacitance will peak at a value only several times larger than the one when no bias is applied and then collapses rapidly to a small value[3-7]. As recommended by compact modelings[7], for the one-sided step junction and the Schottky junction, the voltage V_p applied on the space charge region when the capacitance will peak is about $V_p=\psi_{bi}-5V_t=0.30$ V, i.e. at that time the potential barrier height at the semiconductor side is about $5qV_t$. When applied voltage further increases and the barrier is lower than $5qV_t$, the capacitance starts to collapse because storage of the free-carrier in the space charge region starts to fail. At V_p, the peak capacitance calculated by the plate-capacitor model is about C_p=28 nF cm^{-2}.

For more details about the relationship between V_p and the corresponding voltage applied to the emitter, in Supplementary Fig. 4b the on-current data of the Gr-Si

junction was fitted using a Schottky junction model without considering series resistance $J=J_0[\exp(V/\eta V_t)-1]$, where the fitted leakage current density is $J_0=3.3\times10^{-5}$ A cm^{-2} and the ideality factor $\eta=1.85$. When the voltage is about 0.20 V, the series resistance starts to affect the current, and the data starts to deviate from the model. When the applied voltage on the space charge region is $V_p=0.30$ V (and a current can be calculated from the model shown in the red dash line), the corresponding voltage applied to the emitter is about 0.33 V (to achieve an equal current seen from the data shown in the black solid line).

Next, the emitter capacitance has been measured experimentally. The C-V curve of the Si-Gr at a forward bias (Gr is connected to the ground) shows that the peak capacitance is about 41 nF cm^{-2} at -0.30 V (measured at a frequency of 100 kHz) (Supplementary Fig. 4c), which is consistent with the above theoretical estimation. Multiple emitters have been measured and the peak capacitance is between 30 and 50 nF cm^{-2}. At the forward bias of -0.30 V, the capacitance-frequency characteristics (Supplementary Fig. 4d) shows that the capacitance is stable up to 100 kHz, and the measurement result is affected by interface and series resistance at frequency beyond 100 kHz[8]. Thus, the frequency used to measure the capacitance is selected to be 100 kHz.

Based on the above analysis, the capacitance used in this paper is the peak capacitance $C_p=41$ nF cm^{-2}, leading to a conservative evaluation of the alpha cut-off frequency. This capacitance is at least one-order-of-magnitude lower than its tunnel emitter counterparts (Supplementary Table 2).

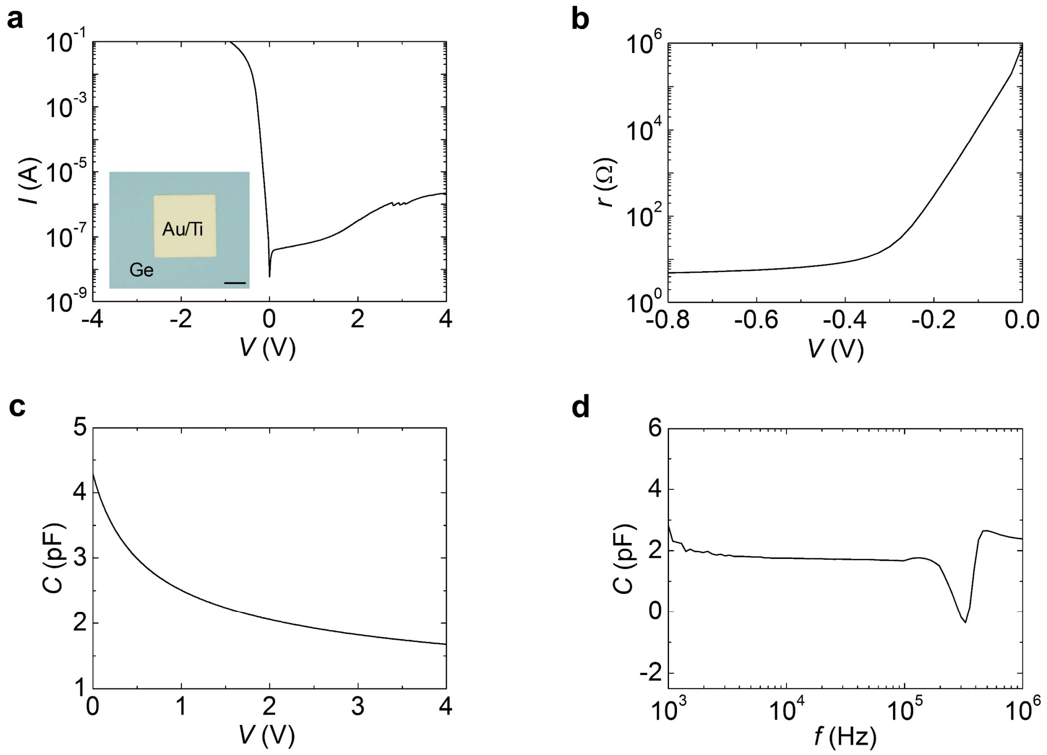

Supplementary Fig. 5 Series resistance and capacitance measurement of a Au/Ti-Ge Schottky junction. **a** I-V characteristic of the junction. The Au/Ti is connected to the ground. Inset: an optical image of the junction (scale bar: 20 μm). **b** Differential resistance *r* calculated from the I-V characteristic at a forward bias, which tends to the series resistance about 5 Ω. **c** The C-V characteristic of the junction at reverse bias at a frequency *f* of 100 kHz. As the reverse bias increases, *C* decreases because the depleted region width increases. **d** The C-f characteristic of the junction at a reverse bias of 4 V. The capacitance is stable up to 100 kHz. 100 kHz is selected.

f_α will be analyzed and estimated in detail as below. Firstly, it should be emphasized that the f_α estimated here is the intrinsic f_α. Only delay time contributed by junction capacitance will be included, and the effect of the parasitic electrode capacitance should be excluded[9]. In our device, the large area Au and graphene on top of the 30-nm-thick Al_2O_3-Ge substrate (Gr/Au-Al_2O_3-Ge) (Fig. 1) will induce large

parasitic capacitance which is orders-of-magnitude larger than the Gr-Ge collector junction capacitance. It is not useful for implementing device functions, and in a device for production, it can be reduced by for example, mesa Ge structure together with thick insulating layer and small base electrode. In the following estimation of f_α, this parasitic capacitance is excluded.

Estimation of τ_e

The intrinsic alpha cut-off frequency f_α can be expressed[10] as $f_\alpha=1/[2\pi(\tau_e+\tau_b+\tau_c)]$. The base transit time τ_b is ignored ($\tau_b=0$) thanks to the atomically thin thickness of graphene. The emitter charging time τ_e is calculated as $\tau_e=C_e/g_e$ where C_e=41 nF cm^{-2} is the peak emitter capacitance (Supplementary Fig. 4c) and g_e is the emitter conductance which can be achieved from the I-V characteristics of the emitter junction (347 S cm^{-2} at −5 V) (Fig. 2a), leading to a $\tau_e=C_e/g_e$=118 ps at −5 V.

Estimation of τ_{cc}

The collector delay time τ_c can be expressed as $\tau_c=\tau_{ct}+\tau_{cc}$ where τ_{ct} is the collector (depleted region) transit time and τ_{cc} is the collector charging time. We will first estimate τ_{cc} with experimental results. τ_{cc} is estimated by $\tau_{cc}=r_cC_c$ where C_c is the collector capacitance and r_c is the collector series resistance. As discussed, to estimate the intrinsic f_α, the effect of the parasitic electrode capacitance should be excluded, thus C_c cannot be directly measured from the Gr-Ge junction in the device, because the result will contain the parasitic electrode capacitance. At the same time, r_c cannot be directly measured from the I-V characteristics of the Gr-Ge junction either, because the measured series resistance of the junction will contain not only the collector series

resistance, but also the base series resistance.

To determine C_c and r_c, we have fabricated Au(50nm)/Ti(5nm)-Ge Schottky junctions (Supplementary Fig. 5a) using the same n^+-Ge substrate as the device in Fig. 3e, where the area of Au/Ti is the same as the Gr-n^+-Ge junction area, i.e. the area of the window of Al_2O_3 (Fig. 1b). We measure the series resistance (Supplementary Fig. 5b) and the capacitance at a reverse bias of 4V (Supplementary Fig. 5c,d) of the Au/Ti-Ge junctions, and these results will be r_c (about 5 Ω) and C_c (1.7 pF), leading to a $\tau_{cc}=r_cC_c$=8.5 ps. The reason is discussed below.

1. The series resistance of the Au/Ti-Ge Schottky junction is the sum of a) the resistance integrated over the quasi-neutral region (between the depletion-layer edge and bottom Au electrode), b) the spreading resistance in the substrate, and c) the resistance due to the bottom Au ohmic contact with the substrate, if the resistance of Au(50nm)/Ti(5 nm) is ignored[2]. For the Gr-Ge junction, the collector series resistance r_c is also the sum of the above 3 items. Since the same Ge substrate with bottom Au electrode is used for the Au-Ge junction and the Gr-Ge junction and the junction areas are also the same, the latter two items (b and c) should be the same for Gr-Ge junction and Au-Ge junction. The width of the depletion-layer is different for the two junctions, but only with a difference less than 1 μm. The thickness of the Ge substrate is about 500 μm, thus the distance between the depletion-layer edge and bottom Au electrode is almost the same, and the first item (a) should also be the same. Based on the above analysis, the measured series resistance of the Au/Ti-Ge Schottky junction is the same as the Ge series resistance in the Gr-Ge junction r_c.

2. At a reverse bias V, the capacitance of a Schottky junction can be calculated[2] as $C=\varepsilon_{Ge}\varepsilon_0/W$, $W=(2\varepsilon_{Ge}\varepsilon_0/qN_d\cdot(\psi_{bi}+V-V_t))^{0.5}$, $\psi_{bi}=\phi_{Bn}-V_n$, $V_n=V_t\cdot\ln(N_c/N_d)$, where ε_{Ge} is the relative dielectric constant of Ge, ε_0 is the permittivity in vacuum, W is the width of the depletion region, q is elementary charge, N_d is the doping concentration of Ge, ψ_{bi} is the potential barrier height at the Ge side when no bias is applied, V_t is the thermal voltage, $q\phi_{Bn}$ is the Schottky barrier height, V_n is the Fermi potential of Ge, and N_c is the effective density of states in conduction band. The same Ge substrate is used for Au-Ge and Gr-Ge junctions, thus the difference of the capacitance comes from ϕ_{Bn} which is 0.52 V for Ti-Ge[11] and 0.22 V for Gr-Ge (Supplementary Fig. 7). However, at a large reverse bias, for example V=4 V, the difference of ϕ_{Bn} (0.30 V) can be ignored, thus the difference of W can be ignored. The measured capacitance of Au/Ti-Ge junction is the intrinsic capacitance C_c of the Gr-Ge collector junction.

Estimation of τ_{ct}

Next, τ_{ct} is estimated[10] by $\tau_{ct}=\chi/(2v)$ where χ is the width of the depletion region of the collector junction and v is saturation velocity in Ge (7×10^6 cm s^{-1}). χ is estimated by $\chi=\varepsilon_{Ge}\varepsilon_0/C_c=3.0\times10^{-5}$ cm where C_c is the measured collector capacitance (1.7 pF or say 4.7×10^{-8} F cm^{-2} at 4 V in Supplementary Fig. 5c), leading to a $\tau_{ct}=\chi/(2v)$=2.1 ps.

Estimation of f_α

Based on the above experimental results and analysis, the intrinsic alpha cut-off frequency $f_\alpha=1/[2\pi(\tau_e+\tau_b+\tau_{cc}+\tau_{ct})]$ at a bias of V_c=4 V has been shown in Fig. 2d. With a bias of V_e=−5 V and V_c=4 V, f_α=1/[2π(118+0+8.5+2.1) ps]=1.2 GHz. For the transistors with tunnel emitters (Fig. 2d), only emitter charging time is used to estimate

their f_α, which may decrease when collector delay time is involved.

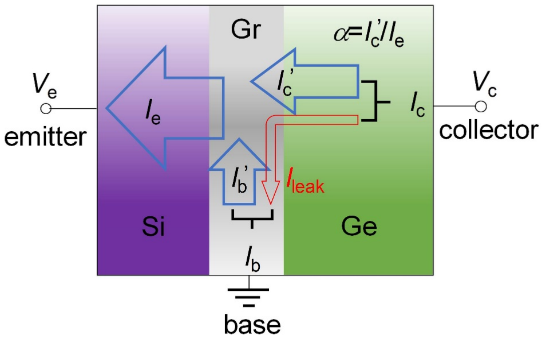

Supplementary Fig. 6 Illustration of the voltage bias and current components for the transistor in the common base mode.

Graphene (Gr) is connected to ground. I_e is emitted from the Si-Gr emitter. A part of I_e is collected at the Ge collector forming the effective collector current I_c', while the other part flows to ground forming I_b'. A leakage current I_{leak} of the collector junction also contributes to the collector current I_c and base current I_b, so that I_c= I_c'+I_{leak}, I_b= I_b'−I_{leak}. For any port (emitter, base, collector), a current I>0 indicates that the current flows into the device and I<0 indicates that the current flows out of the device. The common base current gain is calculated as α=I_c'/I_e.

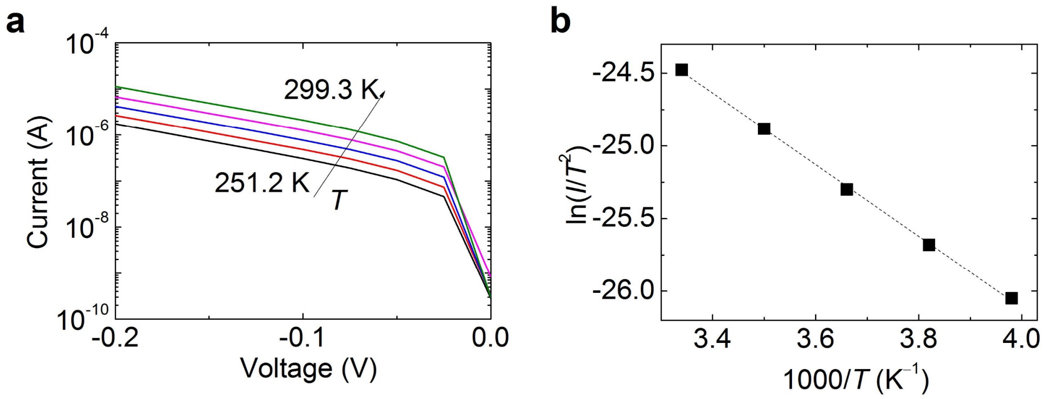

Supplementary Fig. 7 Temperature-dependent I-V characteristics of a Gr-n-Ge Schottky junction. **a** I-V characteristics at the temperatures of 251.2, 261.7, 273.2, 285.6 and 299.3 K. **b** The Schottky barrier height was fitted to be $q\phi_{Bn}$=0.26 eV at a small forward bias of 0.1 V (−0.1 V at n-Ge side) which is lower than that of the Si-Gr emitter junction. A Schottky barrier height of 0.22 eV at a voltage of 0 V is achieved by an extrapolation method (Supplementary Fig. 3).

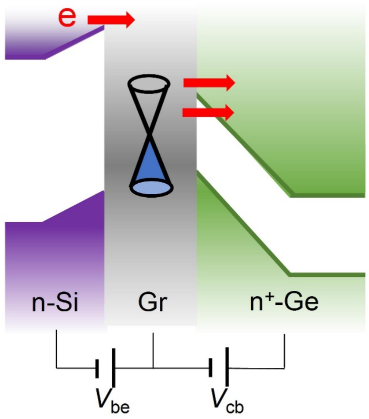

Supplementary Fig. 8 Energy band diagram of the Si-Gr-Ge transistor with a heavily-doped n^+-Ge collector.

The heavily-doped n^+-Ge collector results in a much-increased electrical field at the collector junction. At the collector junction interface, the tunneling distance of an electron decreases dramatically around the top of the barrier. Electrons which cannot cross the collector barrier can tunnel through the barrier, which increases the current gain.

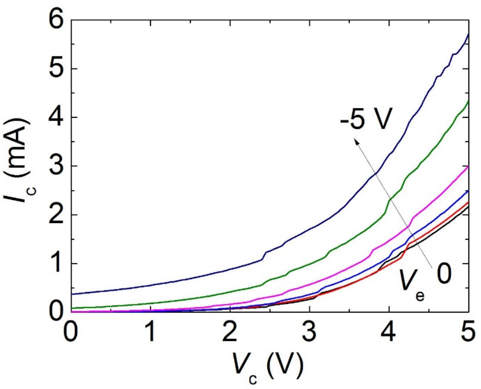

Supplementary Fig. 9 Output characteristics (I_c-V_c) of the transistor with a heavily-doped n^+-Ge collector with V_e from 0 to −5 V.

The output conductance $g_c=dI_c/dV_c$ can be calculated from the output characteristics, and the input conductance $g_e=dI_e/dV_e$ as well as the current gain $\alpha=dI_c/dI_e$ can be calculated from the input characteristics (Fig. 3f). The power gain can thus be expressed as $A_p=(dI_c\cdot dV_c)/(dI_e\cdot dV_e)=(dV_c/dI_c)\cdot(dI_e/dV_e)\cdot(dI_c/dI_e)^2=g_e/g_c\cdot\alpha^2$. When V_c=3 V and V_e=−5 V, the power gain A_p=1.5; and when V_c=3 V and V_e=−4 V, the power gain A_p=1.6. Therefore, the region with power gain larger than 1 can be found.

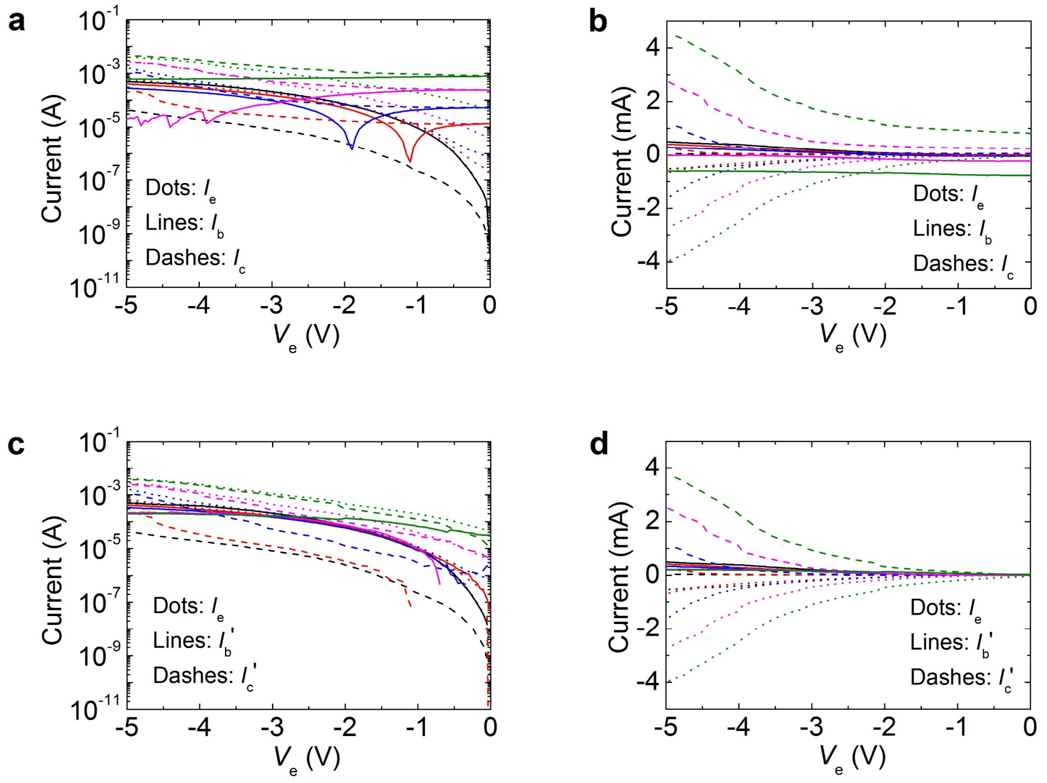

Supplementary Fig. 10 Current components in the transistor with an n$^+$-Ge collector in the common base mode: base current I_b with corresponding emitter current I_e and collector current I_c in **a** the logarithmic coordinates and **b** the linear coordinates, and effective base current I_b' with corresponding emitter current I_e and effective collector current I_c' in **c** the logarithmic coordinates and **d** the linear coordinates. Data for different collector bias V_c is denoted by different colors: black, red, blue, pink and green are for V_c=0, 1, 2, 3, 4 V respectively.

As V_e increases from 0 to −5 V, I_e increases and I_c', I_b' increases correspondingly. I_c= I_c'+I_{leak} also increases, where I_{leak} is the leakage current at the collector junction. I_b= I_b'−I_{leak} tends to change the current direction (from <0 to >0) as I_b' increases and surpasses I_{leak}. A current I>0 indicates that the current flow into the device and I<0 indicates that the current flow out of the device. The above characteristics indicate a

functional device.

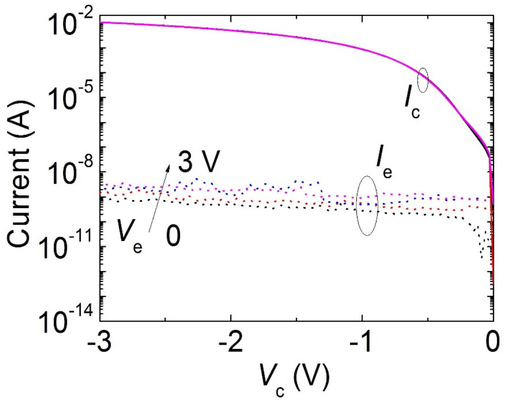

Supplementary Fig. 11 I-V characteristics of the transistor in which the emitter and collector are exchanged. The electrons are emitted from n^+-Ge (V_c<0) and collected at Si (V_e >0). The current and voltage applied at the Ge (Si) side is denoted as V_c (V_e) and I_c (I_e), respectively. The collected current I_e is limited because the barrier height of the Si-Gr junction is higher than that of the Gr-Ge junction. There is no obvious leakage between Si and Ge. In addition, I_c is independent of V_e. Therefore, all these results indicate that the emitter and the collector are not in direct contact.

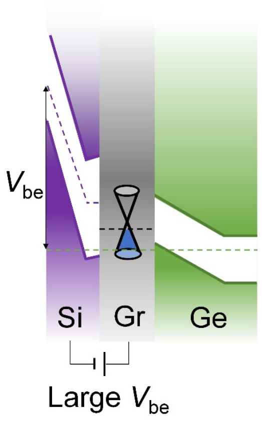

Supplementary Fig. 12 Energy band diagram of the Si-Gr-Ge transistor when a large bias V_{be} applied to the emitter junction.

When a large bias V_{be} is applied on the emitter junction, the current will be limited by the series resistance, and most of the bias is applied to the series resistance of the junction[2]. The series resistance starts to affect the current at about 0.20 V (Supplementary Fig. 15).

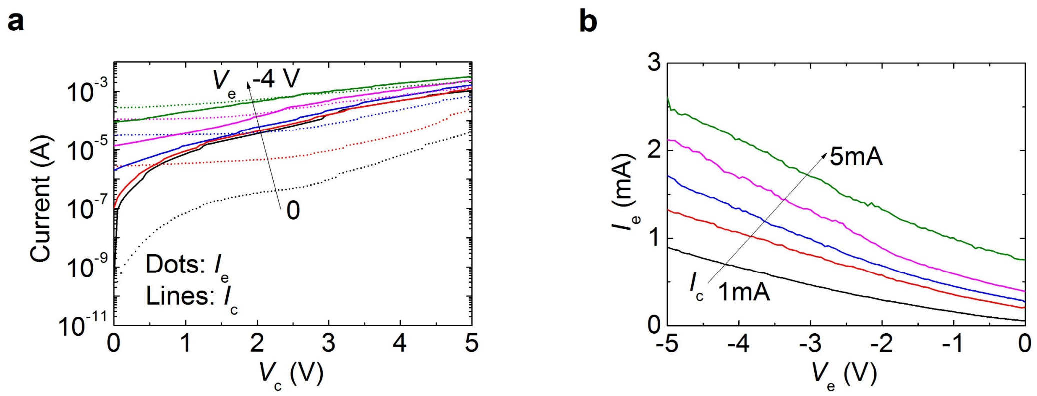

Supplementary Fig. 13 Electrical performance of the transistor with a heavily-doped n$^+$-Ge collector in a reverse working mode. **a** Input (I_c-V_c) and transfer (I_e-V_c) characteristics. **b** Output (I_e-V_e) characteristics.

Based on the quantum capacitance effect of graphene, a reverse working mode of the transistor was studied. The transistor was in the common base mode. Graphene was connected to ground. Ge was used as input with V_c>0 and Si was used as output with V_e<0. As shown in the input (I_c-V_c) and transfer (I_e-V_c) characteristics, when V_c increases, I_c increases, and I_e increases accordingly because of the quantum capacitance effect of graphene (Fig. 4c). This effect can also be observed in the output characteristics. The highest current gain dI_e/dI_c is about 77% when V_c=1.55 V and V_e=–4 V.

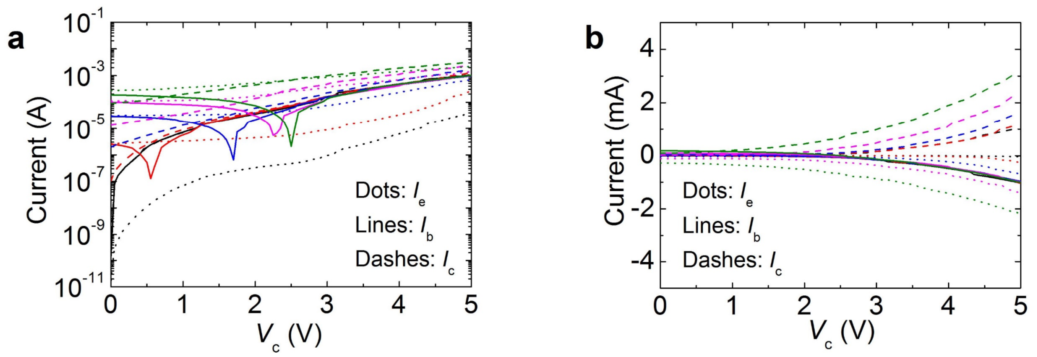

Supplementary Fig. 14 Base current I_b with the corresponding emitter current I_e and collector current I_c in **a** the logarithmic coordinates and **b** the linear coordinates for the transistor in the reverse working mode (Supplementary Fig. 13). Data for different collector bias V_e is denoted by different colors: black, red, blue, pink and green are for V_e=0, −1, −2, −3, −4 V, respectively.

As V_c increases from 0 to 5 V, I_c increases and I_e increases accordingly. The leakage current I_{leak} at the collector junction also increases, and I_b= I_b'−I_{leak} tends to change the current direction (from >0 to <0) as I_{leak} increases and surpasses I_b'. Here I_b' is effective base current. Current components are illustrated in Supplementary Fig. 6. A current I>0 indicates that the current flow into the device and I<0 indicates that the current flow out of the device.

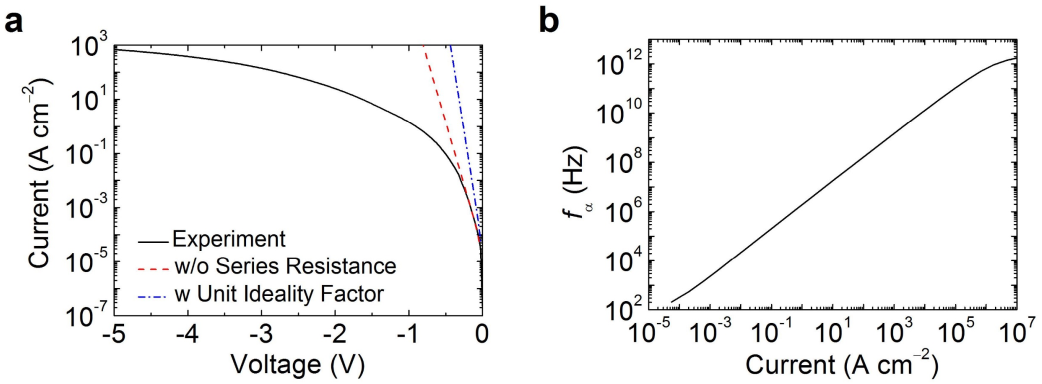

Supplementary Fig. 15 Terahertz operation using the Si-Gr emitter in an ideal case. **a** The I-V characteristics of the Si-Gr emitter obtained from experiments (black solid line), and those fitted using a Schottky junction model without considering series resistance (red dashed line) and further using an ideality factor of 1 (blue dash-dot line). **b** When a heavily-doped Si emitter and a thin collector are used, the ideal Si-Gr emitter without series resistance and with ideal interface shows a cut-off frequency of 1 THz at 2.1×10^6 A cm^{-2}.

The experimental data (black solid line) of the I-V characteristics of the Si-Gr emitter was fitted using a Schottky junction model without considering series resistance $J=J_0[\exp(V/\eta V_t)-1]$, where the fitted leakage current density is $J_0=3.3\times10^{-5}$ A cm^{-2}, the ideality factor $\eta=1.85$ and V_t is the thermal voltage (red dashed line). When the voltage is larger than 0.2 V, the model deviates from the experimental data because of the series resistance. An ideal case was considered. The series resistance was ignored and an ideal interface with unit ideality factor $\eta=1$ was further assumed (blue dash-dot line), which further increases the current and conductance.

A heavily-doped n^+-Si emitter was used in this estimation (doping concentration: 8×10^{19} cm^{-3}). The I-V characteristics can be predicted by the model of the ideal lightly-

doped Si emitter case (blue dash-dot line in Supplementary Fig. 15 a), if the same Schottky barrier height $q\phi_{Bn}$=0.68 eV at the graphene side is assumed which is determined by experiment (Supplementary Fig. 3 and Fig. 2b). The conductance g_e can thus be estimated from the I-V characteristics. The capacitance C_e was calculated from the plate-capacitor model as $C_e=\varepsilon\varepsilon_0/W$, $W=(2\varepsilon\varepsilon_0/qN_d\cdot(\psi_{bi}-V-V_t))^{0.5}$, $\psi_{bi}=\phi_{Bn}+V_n$, $V_n=V_t\cdot(\ln(N_d/N_c)+2^{-3/2}\cdot(N_d/N_c))$, where ε is the relative dielectric constant, ε_0 is the permittivity in vacuum, W is the width of the depletion width for Si, q is elementary charge, N_d is the doping concentration of Si of 8×10^{19} cm^{-3}, $q\psi_{bi}$ is the potential barrier height at the Si side when no bias is applied, V_t is the thermal voltage, $q\phi_{Bn}$ is the Schottky barrier height of ~0.68 eV, V_n is the Fermi potential of the Si, and N_c is the effective density of states in conduction band of Si. The emitter charging time τ_e can thus be calculated as $\tau_e=C_e/g_e$. A small collector delay time τ_c can be achieved by heavily-doped semiconductors[12] or thin collector semiconductors such as multilayer 2D materials[13,14]. Assuming a thickness of χ=5 nm and a saturation velocity[15] $v=4\times10^6$ cm s^{-1} gives the collector delay time[2] $\tau_c=\chi/(2v)=6.25\times10^{-14}$ s. The alpha cut-off frequency f_α was estimated by $f_\alpha=1/(2\pi(\tau_e+\tau_c))$ with ignored base transit time τ_b. With a current of about 2.1×10^6 A cm^{-2}, f_α of about 1 THz is obtained (Supplementary Fig. 15b), which is consistent with the theoretical predictions, demonstrating that the graphene-base heterojunction transistor has a potential application in the THz operation[12].

Overall, the gap between theoretical and experimental results are partly induced by the contact (increasing series resistance) and interface quality (increasing series resistance and ideality factor), which can be improved by contact engineering (such as

using heavily-doped semiconductor and ion implantation) and interface engineering (Supplementary Discussion 1). A heavily-doped emitter is needed for higher current and conductance, and a heavily-doped collector or a thin 2D material collector should also be used to reduce the collector delay time.

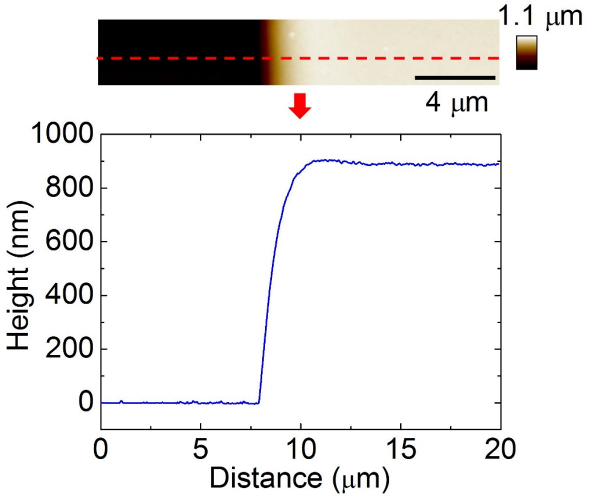

Supplementary Fig. 16 Height of a Si membrane (without the Au electrode) measured by AFM. The typical height of a Si membrane is ~880 nm.

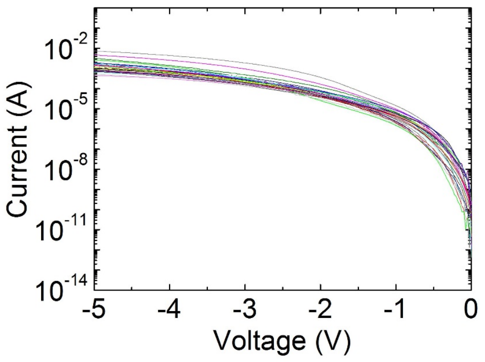

Supplementary Fig. 17 I-V characteristics of the Si-Gr emitters of 28 Si-Gr-Ge transistors in one batch.

Supplementary Table 1 Comparison of Schottky barrier height ($q\phi_{Bn}$) and ideality factor of graphene/n-Si Schottky diodes.

Reference	$q\phi_{Bn}$ (eV)	Ideality factor	Si doping	Notes
[16]	0.86	1.2-5	2-6×10^{15} cm^{-3}	
[17]	0.62	1.08	1×10^{16} cm^{-3}	CVD graphene on etched Cu
[17]	0.57	1.5	1×10^{16} cm^{-3}	-
[18]	0.69	1.46	1×10^{15} cm^{-3}	exfoliated graphene
[18]	0.83	2.53	1×10^{15} cm^{-3}	CVD graphene
[19]	0.79	1.6-2	0.8-1×10^{15} cm^{-3}	-
[19]	0.89	1.3-1.5	0.8-1×10^{15} cm^{-3}	Graphene doped with TFSA
[20]	0.833-0.858	1.56-1.58	0.05-0.2 Ω cm	-
[21]	0.79	1.41	5×10^{14} cm^{-3}	-
[22]	0.71	3.7	1-10 Ω cm	-
[23]	0.407	1.1	1×10^{16} cm^{-3}	-
This work	0.68	1.85	1×10^{15} cm^{-3}	-

The Si-membrane-on-Gr junction with an ideality factor of ~1.85 and a Schottky barrier height of ~0.68 eV (this work) is benchmarked against those in literature[16-24]. The data are consistent with the reported results.

Supplementary Table 2 Parameters and capacitances for different dielectric layer.

	AlN[14]	SiO_2[25]	$TmSiO_2$ - TiO_2[26]	SiO_2[27]
ε	9	3.9	12	3.9
W (nm)	3	5	6.5	8
C_e (F cm^{-2})	2.66×10^{-6}	6.91×10^{-7}	1.63×10^{-6}	4.32×10^{-7}

The capacitance was calculated as $C_e=\varepsilon\varepsilon_0/W$, where ε is the relative dielectric constant, ε_0 is the permittivity in vacuum, W is the width of the insulator in the tunnel junction.

Supplementary Discussion 1 Strategy of the transistor performance improvement.

A trade-off exists between a higher output impedance using a lightly-doped collector and a larger current gain using a heavily-doped one. A fundamental solution to obtain both high output impedance and large current gain is to improve the quality of the interfaces to reduce interface scattering considering the following issues: 1) graphene growth for a clean bottom surface[17], 2) ultra-clean graphene transfer for a clean top surface, 3) a new synthesis method such as direct graphene synthesis on the Ge substrate[28], and 4) an all 2D material semiconductor-graphene-semiconductor transistor to reduce interface dangling bond scattering and process contamination.

The leakage current of the collector junction could be reduced by the following. 1) Improving the quality of the interfaces as already discussed above to get both a high output impedance and a large current gain without using a heavily-doped collector. 2) Using various techniques for leakage reduction in Ge junctions[29]. 3) Selecting a semiconductor that forms a larger barrier with graphene than Ge as the collector, and another semiconductor which forms an even larger barrier as the emitter. The choices of 2D materials are many and are still growing. A combination of 2D materials may reduce the leakage.

Supplementary Discussion 2 Analysis of electrical characteristics using energy band diagrams.

Two phenomena can be understood based on the results in Fig. 4b. One is that even when no bias is applied to the collector junction, there is a collector current I_c (the black solid lines in Fig. 3b and f) since the injected electrons cause a Fermi energy level difference between graphene and Ge. The other is that the current gain for the transistor with an n^+-Ge collector increases with V_e in Fig. 3g, since an increased V_{be} increases the energy difference between Si and Ge, which makes the Ge energy band move down. At the collector junction interface, around the top of the barrier, the tunneling distance of an electron decreases resulting in an increased tunneling current. This is more obvious for the n^+-Ge collector because the tunneling current for the n-Ge collector is weak.

Another two phenomena can be understood based on Fig. 4c. One is as V_c increases, the current gain in Fig. 3g significantly increases since more emitted electrons can tunnel through the collector barrier. The other is that I_e increases with V_c even at V_e=0 for a transistor with an n^+-Ge collector in Fig. 3f. When V_c is applied, the Fermi energy level of Si moves up, and the pumped-out electrons cause a Fermi energy level difference between Si and Gr. I_e is inversely proportional to $q\phi_{Si}$, as shown in Fig. 4c when there is a Fermi energy level difference between Si and Gr.

Supplementary References

1. Schroder, D. K. *Semiconductor Material and Device Characterization, Third Edition* (John Wiley & Sons, Inc., New Jersey, 2006).
2. Sze, S. M. & Ng, K. K. *Physics of Semiconductor Devices, Third Edition* (John Wiley & Sons, Inc., New Jersey, 2007).
3. Bouma B. C. & Roelofs A. C. An experimental determination of the forward-biased emitter-base capacitance. *Solid-State Electron.* **21**, 833-836 (1978).
4. Hjelmgren, H., Kollberg E. & Lundgren, L. Numerical simulations of the capacitance of forward-biased Schottky-diodes. *Solid-State Electron.* **34**, 587-590 (1991).
5. Lee S. W. & Prendergast E. J. Analytical relations for determining the base transit times and forward-biased junction capacitances of bipolar transistors. *Solid-State Electron.* **28**, 767-773 (1985).
6. Liou, J. J. & Malocha, D. C. Modeling the non-quasi-static metal-semiconductor space-charge-region capacitance. *J. Appl. Phys.* **65**, 1782-1787 (1989).
7. Liou, J. J. & Lindholm, F. A. Forward-voltage capacitance and thickness of p-n junction space-charge regions. *IEEE Trans. Electron Dev.* vol. **ED-34**, 1571-1579 (1987).
8. Şahin, B., Çetin, H. & Ayyildiz, E. The effect of series resistance on capacitance–voltage characteristics of Schottky barrier diodes. *Solid State Commun.* **135**, 490-495 (2005).
9. Liao, Lei et al. High-speed graphene transistors with a self-aligned nanowire gate.

Nature **467**, 305-308 (2010).

10. Atalla, M. M. & Soshea, R. W. Hot-carrier triodes with thin-film metal base. *Solid-State Electron.* **6**, 245-250 (1963).
11. Nishimura, T., Kita, K. & Toriumi, A. Evidence for strong Fermi-level pinning due to metal-induced gap states at metal/germanium interface. *Appl. Phys. Lett.* **91**, 123123-1-3 (2007).
12. Di Lecce, V. et al. Graphene-base heterojunction transistor: an attractive device for terahertz operation. *IEEE Trans. Electron Dev.* **60**, 4263-4268 (2013).
13. Guo, H. et al. All-two-dimensional-material hot electron transistor. *IEEE Electron Dev. Lett.* **39**, 634-637 (2018).
14. Zubair, A. et al. Hot electron transistor with van der Waals base-collector heterojunction and high-performance GaN emitter. *Nano Lett.* **17**, 3089-3096 (2017).
15. Jin, Z., Li, X., Mullen, J. T. & Kim, K. W. Intrinsic transport properties of electrons and holes in monolayer transition-metal dichalcogenides. *Phys. Rev. B* **90**, 045422-1-7 (2014).
16. Tongay, S. et al. Rectification at graphene-semiconductor interfaces: zero-gap semiconductor based diodes, *Phys. Rev. X* **2**, 011002-1-10 (2012).
17. Sinha, D. & Lee, J. U. Ideal graphene/silicon Schottky junction diodes. *Nano Lett.* **14**, 4660-4664 (2014).
18. Parui, S. et al. Temperature dependent transport characteristics of graphene/n-Si diodes. *J. Appl. Phys.* **116**, 244505-1-5 (2014).

19. Miao, X.H. et al. High efficiency graphene solar cells by chemical doping, *Nano Lett.* **12**, 2745-2750 (2012).

20. Shi, E. et al. Colloidal antireflection coating improves graphene-silicon solar cells, *Nano Lett.* **13**, 1776-1781 (2013).

21. Kim, H.-Y., Lee, K., McEvoy, N., Yim, C. & Duesberg, G.S. Chemically modulated graphene diodes. *Nano Lett.* **13**, 2182-2188 (2013).

22. Singh, A., Uddin, M. A., Sudarshan, T. & Koley, G. Tunable reverse-biased graphene/silicon heterojunction Schottky diode sensor. *Small* **10**, 1555-1565 (2014).

23. Yang, H. et al. Graphene barristor, a triode device with a gate-controlled Schottky barrier. *Science* **336**, 1140-1143 (2012).

24. Di Bartolomeo, A. Graphene Schottky diodes: an experimental review of the rectifying graphene/semiconductor heterojunction. *Phys. Reports* **606**, 1-58 (2016).

25. Vaziri, S. et al. A graphene-based hot electron transistor. *Nano Lett.* **13**, 1435-1439 (2013).

26. Vaziri, S. et al. Bilayer insulator tunnel barriers for graphene-based vertical hot-electron transistors. *Nanoscale* **7**, 13096-13104 (2015).

27. Zeng, C. et al. Vertical graphene-base hot-electron transistor. *Nano Lett.* **13**, 2370-2375 (2013).

28. Lee, J. H. et al. Wafer-scale growth of single-crystal monolayer graphene on reusable hydrogen-terminated germanium. *Science* **344**, 286-289 (2014).

29. Liu, T. C., Ikegaya, H., Nishimura, T. & Toriumi, A. Ge n^+/p Junctions with high

on-to-off current ratio by surface passivation. *IEEE Electron Dev. Lett.* **37**, 847-850 (2016).
